# Relationship between body temperature and behavior of nonpregnant early-lactation dairy cows

**DOI:** 10.3168/jdsc.2022-0327

**Published:** 2023-03-02

**Authors:** Maria Elisa Montes, Mercedes Brunton, Adrianna Mann, Kelsey Teeple, Uduak George, Jacquelyn Boerman, Theresa Casey

**Affiliations:** 1 Department of Animal Sciences, Purdue University, West Lafayette, IN 47907; 2 Department of Mathematics and Statistics, San Diego State University, San Diego, CA 92182-7720

## Abstract

Animal behavior and management factors that influence behavior affect physiology and lactation performance. Circadian rhythms of core body temperature are a primary output of the master clock; however, core body temperature in early-lactation dairy cows showed poor fit to 24-h rhythms. We hypothesized that eating behavior was related to daily body temperature oscillations. The objectives of this study were to determine if oscillations in daily behaviors, specifically feeding behavior, were related to body temperature. The behavior of 11 Holstein cows (34 ± 14 d in milk; mean ± standard deviation) housed in a freestall barn was recorded every 10 min for a 48-h period. Simultaneously, data loggers (iButtons; iButtonLink Technology) recorded the body temperature of cows with the same sampling frequency. The mean temperature of all cows showed a better fit to a 2-component cosinor (R^2^ = 0.54) than to a single cosinor model (R^2^ = 0.26). Logistic regression showed that the probability (Pr) of a cow experiencing an increase in body temperature (increment, I) given that she was milking [Pr(I|milking) = 0.94] was higher than for ruminating [Pr(I|ruminating) = 0.69], lying [Pr(I|lying) = 0.66], feeding [Pr(I|feeding) = 0.16], standing [Pr(I|standing) = 0.54], and mounting [Pr(I|mounting) = 0.62]. The main limitations of this study are the length of the observation period and the sample size. Longer observation windows on core body temperature would allow to isolate the noise and the signal and identify patterns with more clarity. Oscillations in body temperature were not associated with feeding. However, findings indicate that milking, activity associated with walking to the parlor, or the temperature in the parlor may affect secondary rhythms of daily body temperature.

## Highlights

• Body temperature oscillations in early-lactation cows were associated with milking.
• Most cows had an increase in body temperature during the milking sessions.
• Feeding, lying, standing, and rumination were not related to body temperature.

## Graphical Abstract

**Figure:**
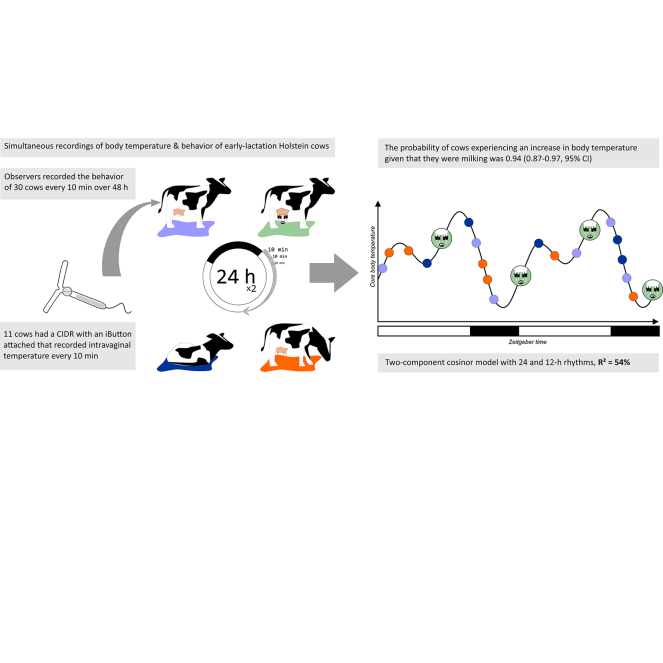
**Summary:** The behavior and core body temperature of 11 early-lactation Holstein cows in a freestall barn were recorded with a frequency of 10 minutes for a 48-hour period. The probability of increment in body temperature given each of the observed behaviors (feeding, lying, standing, mounting, and rumination) was analyzed with a generalized mixed model. The results showed that body temperature oscillations in early-lactation cows were associated with milking, but not with any of the other observed behaviors. The rhythmicity of body temperature was analyzed using cosinor models. Body temperature in early-lactation cows milked twice daily was best described by a 2-component cosinor model that included a circadian rhythm and a 12-hour ultradian rhythm, with the 2 peaks coincident with milking time. Together, these findings indicate that the schedule of milking sessions can be an important input for establishing daily rhythms of body temperature and activities of early-lactation cows.

Regular behavior patterns function as inputs to circadian clocks, and thus everyday activities on a dairy farm, such as feed delivery or milking, can potentially affect a cow's circadian rhythms. Circadian rhythms are 24-h cycles of behavior and physiology that enable animals to anticipate regular changes in their environment. The function of the circadian clocks is to gather time information from external and internal signals and generate rhythms that orchestrate biochemical, physiological, and behavioral events, ensuring the proper timing of processes (Casey et al., 2022). Light is the primary input to the master clock located in the suprachiasmatic nuclei (**SCN**). The SCN converts light information into circadian rhythms of core temperature and hormonal rhythms such as melatonin that communicate the time with the rest of the clocks throughout the body (Buhr et al., 2010). Because robust circadian rhythms are central to maintaining homeostasis, knowledge of the biology underlying circadian clock timing is critical to maximizing dairy cows' health and production efficiency.

Although SCN controls the daily rhythm of core body temperature (Buhr et al., 2010), factors that function as inputs to other circadian clocks can influence body temperature. Reproductive state (Schrader et al., 2009), photoperiod (Kalyesubula et al., 2021), time of feed delivery (Salfer and Harvatine, 2020), physical activity (Verwey and Amir, 2009), stress, and disease (Wagner et al., 2021) can modify or disrupt the daily rhythms of body temperature. Therefore, oscillations or changes in the temperature cycles can be indicators of these events.

Dairy cattle exhibit daily rhythms on feeding, rumination, and rumen pH (DeVries et al., 2003; Salfer et al., 2019). As ruminants, cows anticipate the feeding time, manifesting changes in activity and temperature (Piccione et al., 2003, 2007; Giannetto et al., 2010; Suarez-Trujillo et al., 2022). Thereby the circadian rhythms of feeding, lying behavior, and core body temperature are influenced by feeding time (Niu et al., 2014).

Metabolic and reproductive systems are highly integrated with the circadian timing system and demonstrate reciprocal regulation. Thus, a mismatch between internal time and environmental cues can cause physiological disorders (Goldbeter, 2008). In a previous study, we found circadian rhythms of core body temperature in dairy cattle were more robust during late gestation than in early lactation (Suarez-Trujillo et al., 2022). Analysis of temperature rhythms in periparturient goats yielded similar findings, where a better fit of a 24-h rhythm was found in late gestation and a much lower fit in the early postpartum period (Kalyesubula et al., 2021).

The decrease in fit of the body temperature to a circadian rhythm in the postparturition time points could be related to the changes in physiology, cues from milking, feeding behavior, and social interactions. Our objectives were to determine (1) if nonpregnant, early-lactation dairy cows in a freestall exhibited circadian rhythms of core body temperature and (2) if oscillations in daily behaviors and body temperature were related. We hypothesized that oscillations in body temperature would be related to feeding.

The study was conducted on February 26 to 28, 2021, at the Dairy Unit of the Purdue Animal Sciences Research and Education Center. All protocols in this study were approved by Purdue University's Institutional Animal Care and Use Committee (protocol no. 2006002057). The study sample consisted of 30 early lactation (34 ± 19.4 DIM; mean ± SD) nonpregnant Holstein cows housed in a freestall. Only the data of 11 cows are presented in this report. Each cow was considered the experimental unit and was assigned a study number, which was affixed to ear tags and painted onto flanks using marking paint. Subjects were restrained into headlocks within the pen for marking and then released.

During the study, the lighting schedule at the barn was 16 h of high lux light and 8 h of low lux light. From 0500 to 2100 h light intensity at the cow's eye level was 282 lx on average. From 2100 to 0500 h, light intensity decreased to 128.5 lx on average. The approximate temperature during the day was 10°C, and at night it was 2.5°C. The cattle were milked twice daily. Cows were moved from freestall to parlor at 0500 h for morning milking and at 1700 h for evening milking. Cows were fed a TMR formulated to meet or exceed their nutritional requirements (NRC, 2001). Fresh feed was delivered daily at 0600 h. Feed was pushed at 1200 h, and before and after milking sessions. Refusals were removed during the morning milking.

A subset of cows (n = 12) was randomly selected to measure body temperature every 10 min over 48 h with a resolution of 1/8°C. Vaginal temperature was recorded using internal temperature loggers (iButton DS1921H-F5#, iButtonLink Technology) secured to a blank controlled internal drug release device (EAZI-Breed CIDR Cattle Insert, Zoetis Inc.). The iButtons were attached to blank CIDR using modeling clay and encased in heat shrink tubing. Loggers were set to start recording at 0600 h on February 26, 2021, and devices were inserted into the cows' vaginas the day before. One cow lost her device during the experiment, leaving a subsample of 11 cows (34 ± 14 DIM; mean ± SD).

Beginning at 0600 h on February 26, 2021, 3 observers recorded behavior of 30 cows from outside of the freestall. Every 10 min for 48 continuous hours the observers recorded individual behaviors according to an ethogram (Table 1). When cows were moved to the parlor, milked, and returned back to freestall, the entire period was recorded as milking. The observation records were converted into binary data (1 and 0) for each behavior. Code 1 was used to indicate the behavior and position that were true for each observation, and 0 indicated that the animal was not exhibiting that behavior or position. For example, in the case of feeding, both feeding and standing were indicated as 1, and the other behaviors were 0.

**Table 1:** Ethogram of cow behaviors

Activity or behavior	Description	Code
Lying	The cow is lying without doing anything. The cow could be sleeping.	0
Lying and ruminating^1^	The cow is lying and chewing cud.	1
Standing	The cow is standing on 4 legs. The cow is not doing any of the other behaviors described (ruminating, eating, defecating, urinating, or milking), but could be walking.	2
Standing and feeding^1^	The cow is standing and chewing cud.	3
Standing and defecating or urinating^1^	The cow is standing and is either defecating or urinating.	4
Standing and feeding^1^	The cow is standing with her head through the feed stall and close to feed; she grasps feed and swallows.	5
Milking^2^	From the moment the cow was moved out of the pen to the milking parlor, until she came back.	6
Mounting	The cow is standing on her rear legs and resting her front legs on the other cow's back.	7
Standing and butting^1^	The cow is standing and swinging her head against another cow.	8
Missing	It is not possible to identify the cow.	Null

1 Joint behaviors comprise the description of both behaviors.

2 During the entire milking event, cows were conditioned to be standing; cows were not observed for other behaviors during this period.

Because activity and behavior are inputs to circadian rhythms, binary activity data were overlaid on the body temperature plots to determine if associations existed. To identify more objectively which activities had an association with an increase in body temperature, records of body temperature and behavior of 11 cows were first arranged chronologically. Consecutive behaviors of the same class were defined as an instance. The difference in body temperature was calculated by subtracting the temperature at the end of an instance from the temperature at the start (ΔT). If the difference in body temperature was greater than zero, it was categorized as an increment, and if it was less than or equal to zero as no increment. A generalized mixed model, logistic regression, from the package lmerTest (Kuznetsova et al., 2017) was used to test the probability (**Pr**) of showing an increment (**I**) given that the cow was standing [only, Pr(I|S)], lying [only, Pr(I|L)], ruminating [standing or laying (Pr(I|R)], feeding [Pr(I|F)], mounting [Pr(I|H)], or milking [Pr(I|M)]. The generalized mixed model included the random effects of behavior (milking, standing, feeding, lying, mounting, and ruminating) and random effects of cow specifying a binomial distribution. The probabilities of increment given each behavior was obtained with the inverse link function for logit and the estimated coefficients (β) for each behavior (Equation [1]).

[1] $$\mathrm{Pr}\left(I|behavior\right)=\frac{e^{\beta }}{1+e^{\beta }}.$$

The temperature behavior plots did not show any obvious relationship between temperature variation and lying, standing, feeding, or ruminating behavior. A consistent pattern for temperature increasing while cows were milking (Figure 1) was found, with most cows (d 1: 8/11 and d 2: 11/11) exhibiting an increase in body temperature during the evening milking sessions. During the morning milking sessions, fewer cows (d 1: 6/11 and d 2: 7/11) had an increment in body temperature. The probability of increment in body temperature given milking was the highest [Pr(I|M) = 0.94, *P* < 0.01], followed by ruminating [Pr(I|R) = 0.69, *P* < 0.01], lying [Pr(I|L) = 0.66, *P* < 0.01], and feeding [Pr(I|F) = 0.16, *P* < 0.01]. The effects of standing [Pr(I|S) = 0.54, *P* = 0.48] and mounting [Pr(I||H) = 0.62, *P* = 0.55] were not statistically significant. Seven out of the 30 cows were observed mounting, and only 3 had temperature data, making the number of observations insufficient to draw conclusions regarding this behavior.

**Figure 1:**
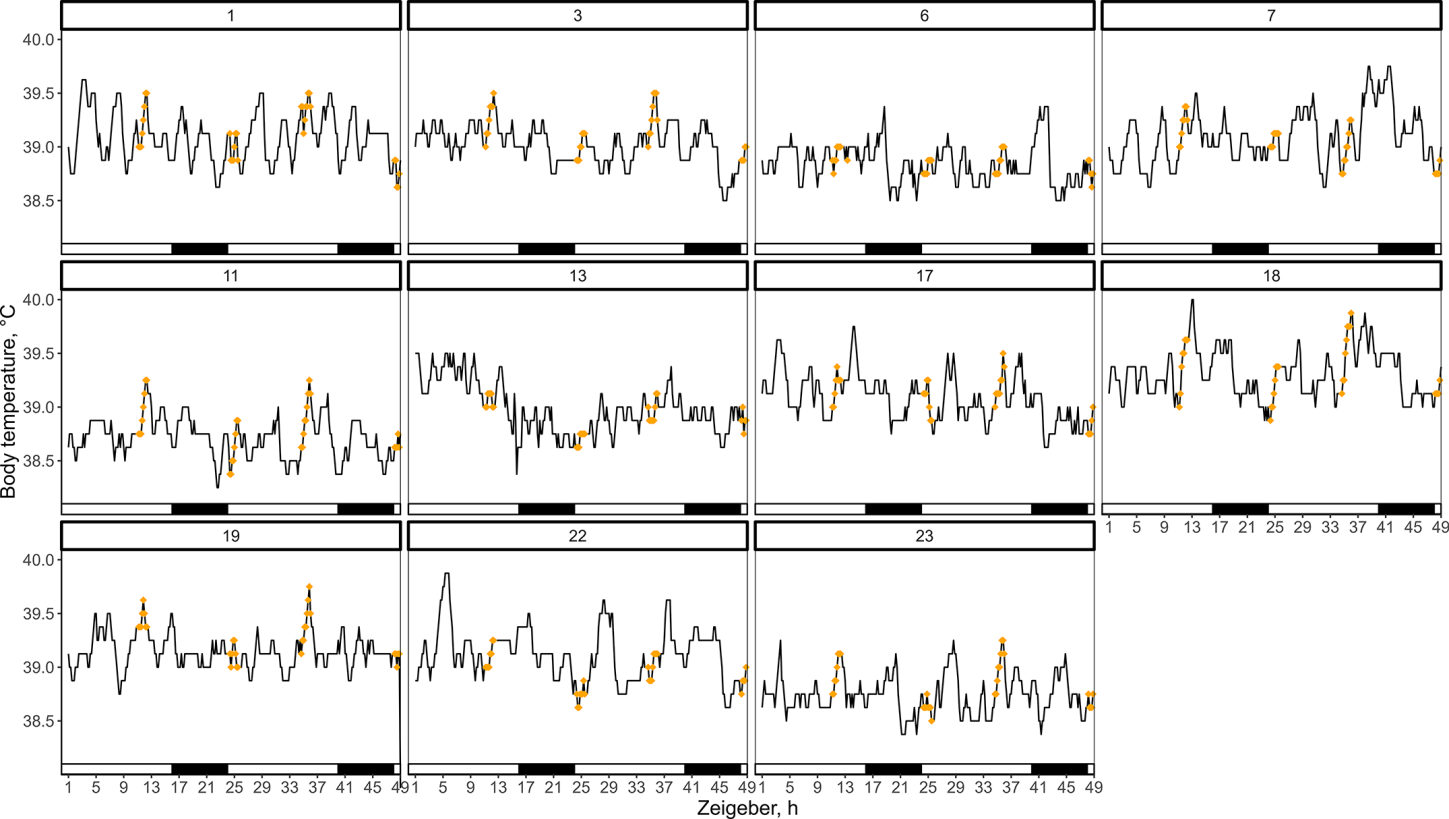
Milking and body temperature (°C) of n = 11 cows with a sampling frequency of 10 min over 48 h. Diamonds indicate when cows were milking. Black squares at the bottom indicate the dark phase.

To evaluate if core body temperature (°C) followed a circadian rhythm, data were analyzed for fit to 1- and 2-component cosinor models using R version 4.1.2 (R Core Team, 2021) and the package card version 0.1.0 (Shah, 2020). A sampling frequency of 10 min over 48 h resulted in 289 observations on the body temperature of each cow. Individual cow records were fitted to individual cosinor models. In addition, the mean of the 11 cows' temperature was used to fit a population cosinor model. For the 1-component cosinor analysis a 24-h period curve was used, and for the 2-component cosinor, an additional curve with a 12-h period was considered. The 1-component and 2-component cosinor can be expressed as shown below in Equation [2] and Equation [3], respectively (Cornelissen, 2014). The MESOR (*M*) is the mean adjusted to the rhythm, the predictable values go from amplitude (*A*) below *M* to amplitude above *M*, acrophase (*ϕ*) is the time between high values, period (*τ*) is the duration of 1 cycle, and *e*(*t*) is the error term.

[2] $$Y\left(t\right)=M+A\cos\;\left(\frac{2\pi t}{\tau }\;+\;\phi \right)+e\left(t\right).$$

[3] $$Y\left(t\right)=M+\overset{2}{\underset{j=1}{\sum }}\;A\cos\;\left(\frac{2\pi t}{\tau_{j}}\;+\;\phi_{j}\right)+e\left(t\right).$$

The *F* statistic and coefficient of determination (R^2^) for the 1- and 2-component cosinor models were estimated based on the equations also described in Cornelissen (2014). The normality of residuals in both population models was assessed with Q-Q plots and Shapiro tests.

The parameters of the circadian and 12-h ultradian rhythm components for the cosinor models of cow body temperature are presented in Figure 2A and 2B. The core body temperature of the cows had a poor fit to a 1-component cosinor with a 24-h period (Figure 2C). The mean R^2^ of individual temperatures was 0.13 ± 0.09 (±SD). The mean body temperature of all cows had a better but still low fit to the 1-component cosinor, R^2^ = 0.26, *P* < 0.01. However, the addition of a second component with a 12-h period improved the adjustment at the individual and population level. The mean R^2^ of the individual temperatures was 0.23 ± 0.17 (±SD), and for the population, R^2^ was 0.54, *P* < 0.01. The 2-component cosinor had 2 peaks within a day, and the inflection points (from negative to positive slope) in this curve were coincident with the time of the milking sessions (Figure 2D).

**Figure 2:**
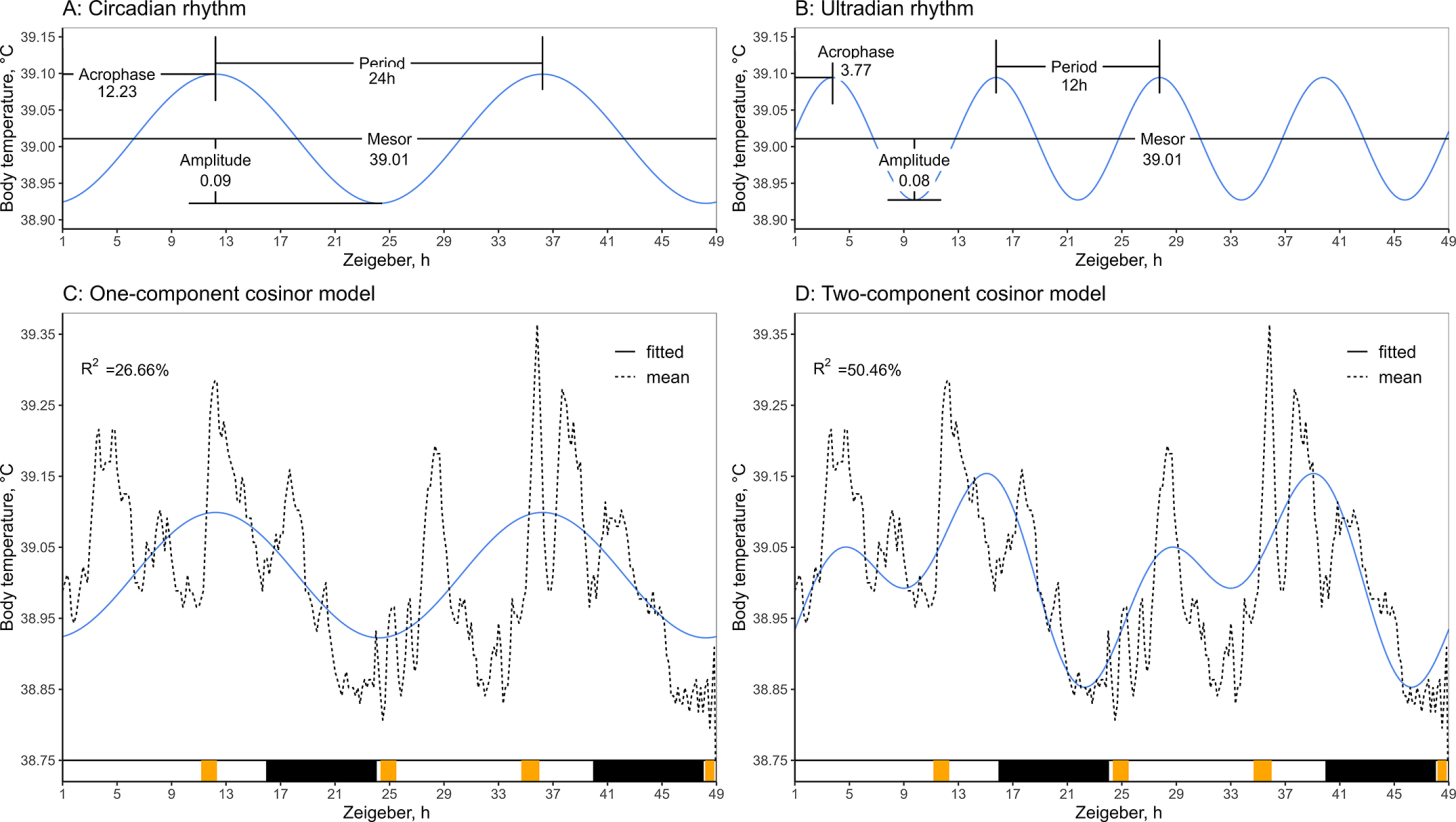
One- and 2-component population cosinor models of the mean body temperature (°C) of n = 11 cows with a sampling frequency of 10 min over 48 h. (A) Parameters of the 24-h rhythm. (B) Parameters of the 12-h rhythm. (C) The 1-component cosinor is a 24-h rhythm. (D) The 2-component cosinor is the sum of the 24-h rhythm and a 12-h rhythm. Black squares indicate the dark phase, and gray squares indicate the milking sessions. The cosinor analysis was conducted with the R package card (Shah, 2020), and the equations are described in Cornelissen (2014).

The data in this trial did not show a relationship between eating behavior and core body temperature of cows in early lactation. Heat is a byproduct of fermentation, and rumen temperature increases soon after ingestion of concentrates (Rutherford et al., 2019). The heat from metabolism affects core body temperature (Finch, 1986), so we hypothesized that the increasing rumen temperature upon ingestion of a TMR would increase body temperature. However, we found no evidence for a temperature increment related to feeding and delivery of feed. This observation is in contrast with the anticipatory feed response we previously noted in late-gestation cows (Suarez-Trujillo et al., 2022) and others reported for lactating cows (Niu et al., 2014). In the current study, the short time relative to parturition may explain why the cows did not show evidence for feed anticipatory response. Clocks of early-lactation dairy cows may still be adjusting to not only the physiological state but also to changes in their environment including feed delivery, milking, and barn light schedules, as well as social interactions within a new herd-cohort. Lack of an association may also be related to the relatively high frequency of eating across the day. The mean number of eating bouts within the 48-h period was 21.9 ± 3.6, with a range of 17 to 29.

There are several potential explanations for the relationship between body temperature and milking period. Milking activity encompassed the time cows were moved from the freestall to the parlor, milking in the parlor and back to the freestall, which took approximately 1 h. Unfortunately, the data captured from the milking parlor software did not record actual time of milking, so the relation of temperature increment to time of milking is unknown. Therefore, the temperature increment can be attributed to increases in body temperature related to exercise (Arave et al., 1987), microenvironment at the holding pen (Nordlund et al., 2019), or milking. Our previous studies of late-gestation, nonlactating cows did not show changes in temperature related to times of exercise (Suarez-Trujillo et al., 2022). The sampling frequency of the previous study was 30 min versus 10 min in the current study, which may have resulted in missing changes in body temperature associated with exercise. Although the climate conditions during the trial were not warm enough to cause heat stress in the cows, the reduced area and airflow in the pre-milking holding pen create a microenvironment in which dissipation of heat by cows may be more difficult than in the freestall (Nordlund et al., 2019). Milking itself may underly the increase in body temperature. Oxytocin is released during milking. The neuroendocrine regulated release of oxytocin is induced by tactile stimulation of the cow's teat in the milking parlor (Bruckmaier and Blum, 1998). Oxytocin is thermoregulatory and exogenous treatment with oxytocin was shown to increase core body temperature in rodents (Camerino, 2021). Future studies will need to examine a larger number of cows and record activities encompassed within this period to determine if milking and associated oxytocin release caused the increment of body temperature observed.

The primary limitations of this study are the length of the observation period and the sample size. Longer observation windows on core body temperature, more frequent sampling, and thermometers with higher accuracy and resolution would allow to isolate the noise and the signal and identify patterns with more clarity. Another limitation is the sampling frequency of behavior. In the current study, behaviors were recorded every 10 min. Still, it is possible that changes in behavior occurring at a greater rate were ignored. Because temperature is a continuous variable, it is possible to interpolate the values within samplings, but that is impossible with behavior that is a categorical variable. Moreover, behavior was not recorded with video or activity loggers, leading to missing data, data entry errors, and potentially observer bias. The timestamps at which each cow began and finished milking could not be recovered because farm backups on the corresponding milking sessions were unavailable. Despite these limitations, this study is interesting and relevant because it shows how not only the behavior but also the physiology of early-lactation cows respond to the routine activities of the dairy farm, leading to further research questions.

An association between the milking event and a rise in core body temperature of nonpregnant cows in early lactation was found. The other observed behaviors were not associated with changes in body temperature under analyses performed in this study. Body temperature in early-lactation cows milked twice daily was best described by a 2-component cosinor model that included a circadian rhythm and a 12-h ultradian rhythm, with the 2 peaks coincident with milking time. Therefore, the schedule of milking appears to be an important input for establishing temperature rhythms and diurnal activities of cows.
